# Supplementary material for: Dynalign II: common secondary structure prediction for RNA homologs with domain insertions
Source: Nucleic Acids Res. 2014 Nov 21;42(22):13939–48. doi: 10.1093/nar/gku1172 (PMC4267632; doi:10.1093/nar/gku1172)
Supplement: SUPPLEMENTARY DATA [file supp_gku1172_nar-02021-z-2014-File012.zip › manual/RNA_class/html/class_multilign__object-members.html]

RNAstructure Classes: Member List

- Main Page
- Classes
- Files

- Class List
- Class Hierarchy
- Class Members

# Multilign\_object Member List

This is the complete list of members for Multilign\_object, including all inherited members.

|  |  |  |
| --- | --- | --- |
| AddOneInput(const string seq, const string ct, const string constraint="", const string shape="") | Multilign\_object |  |
| aliFiles | Multilign\_object | `[private]` |
| AverageLength() const | Multilign\_object |  |
| CleanupIntermediateFiles() const | Multilign\_object |  |
| CountBP(const int i=0, const int j=0, const double percent=0.8) const | Multilign\_object |  |
| ct\_files | Multilign\_object | `[protected]` |
| dGIndex | Multilign\_object | `[protected]` |
| dsvFiles | Multilign\_object | `[private]` |
| energies | Multilign\_object | `[protected]` |
| ErrorCode | Multilign\_object | `[protected]` |
| get\_dGIndex() | Multilign\_object | `[inline]` |
| get\_energies() | Multilign\_object | `[inline]` |
| get\_pair\_alignments() | Multilign\_object | `[inline]` |
| GetErrorCode() const | Multilign\_object | `[inline]` |
| GetErrorMessage(const int error) const | Multilign\_object |  |
| GetIndexSeq() const | Multilign\_object |  |
| GetInputFilenames() | Multilign\_object |  |
| GetIterations() const | Multilign\_object |  |
| GetMaxDsv() const | Multilign\_object |  |
| GetMaxPairs() const | Multilign\_object |  |
| GetNucType() const | Multilign\_object |  |
| GetPairs() | Multilign\_object |  |
| GetProgress() const | Multilign\_object |  |
| GetSequenceNumber() const | Multilign\_object |  |
| GetSHAPEIntercept() const | Multilign\_object |  |
| GetSHAPESlope() const | Multilign\_object |  |
| GetTemperature() const | Multilign\_object |  |
| input\_alignment | Multilign\_object | `[protected]` |
| input\_sequences | Multilign\_object | `[protected]` |
| inputList | Multilign\_object | `[private]` |
| instance | Multilign\_object | `[private]` |
| isRNA | Multilign\_object | `[private]` |
| iterations | Multilign\_object | `[private]` |
| maxDsv | Multilign\_object | `[private]` |
| maxPairs | Multilign\_object | `[private]` |
| Multilign\_object() | Multilign\_object |  |
| Multilign\_object(const vector< vector< string > > &inputlist, const bool isrna=true, TProgressDialog \*progress=NULL) | Multilign\_object |  |
| Multilign\_object(const bool Multifind, const string &outputmultifind, const vector< string > &ctfiles, TProgressDialog \*progress=NULL, const bool isrna=true) | Multilign\_object |  |
| MultiTempMultilign() | Multilign\_object |  |
| NameAliFiles() | Multilign\_object | `[private]` |
| NameDsvFiles() | Multilign\_object | `[private]` |
| NameMultifindAliFiles() | Multilign\_object | `[private]` |
| NameMultifindDsvFiles() | Multilign\_object | `[private]` |
| output\_multifind | Multilign\_object | `[protected]` |
| pair\_alignments | Multilign\_object | `[mutable, protected]` |
| PairMultifindSeq1() | Multilign\_object | `[private]` |
| PairSeq1() | Multilign\_object | `[private]` |
| PrepInput() | Multilign\_object | `[private]` |
| PrepMultifindInput() | Multilign\_object | `[private]` |
| progress | Multilign\_object | `[private]` |
| ProgressiveMultilign(const short int numProcessors=1, const bool Dsv=1, const bool Ali=1, const short int maxtrace=750, const short int bpwin=2, const short int awin=1, const short int percent=20, const short int imaxseparation=-99, const float gap=0.4, const bool singleinsert=true, const short int singlefold\_subopt\_percent=30, const bool local=false) | Multilign\_object |  |
| Randomize() | Multilign\_object |  |
| RemoveOneInput(const string seq) | Multilign\_object |  |
| seqPair | Multilign\_object | `[private]` |
| SetIndexSeq(size\_t indexSeq=1) | Multilign\_object |  |
| SetIndexSeq(const string seqname) | Multilign\_object |  |
| SetIterations(const int it=2) | Multilign\_object |  |
| SetMaxDsv(const float maxdsvchange=1) | Multilign\_object |  |
| SetMaxPairs(const int maxpairs=-1) | Multilign\_object |  |
| SetNucType(const bool isrna=true) | Multilign\_object |  |
| SetProgress(TProgressDialog \*Progress=NULL) | Multilign\_object |  |
| SetSHAPEIntercept(const double intercept=-0.6) | Multilign\_object |  |
| SetSHAPESlope(const double slope=1.8) | Multilign\_object |  |
| SetTemperature(const double temp=310.15) | Multilign\_object |  |
| SHAPEIntercept | Multilign\_object | `[private]` |
| SHAPESlope | Multilign\_object | `[private]` |
| StopProgress() | Multilign\_object |  |
| temperature | Multilign\_object | `[private]` |
| ToHead(vvs\_it first, vvs\_it middle) | Multilign\_object | `[private]` |
| vs\_index typedef | Multilign\_object | `[private]` |
| vvs\_cit typedef | Multilign\_object | `[private]` |
| vvs\_it typedef | Multilign\_object | `[private]` |
| WriteAlignment(const string allali="all.ali") const | Multilign\_object |  |
| ~Multilign\_object() | Multilign\_object |  |

---

Generated on Tue Jul 30 10:52:29 2013 for RNAstructure Classes by 
 1.5.7.1
